# Development of Spontaneous Activity in the Avian Hindbrain

**DOI:** 10.3389/fncir.2016.00063

**Published:** 2016-08-12

**Authors:** Yoko Momose-Sato, Katsushige Sato

**Affiliations:** ^1^Department of Nutrition and Dietetics, College of Nutrition, Kanto Gakuin UniversityYokohama, Japan; ^2^Department of Health and Nutrition Sciences, Faculty of Human Health, Komazawa Women’s UniversityTokyo, Japan

**Keywords:** optical recording, spontaneous activity, chick embryo, brainstem, development

## Abstract

Spontaneous activity in the developing central nervous system occurs before the brain responds to external sensory inputs, and appears in the hindbrain and spinal cord as rhythmic electrical discharges of cranial and spinal nerves. This spontaneous activity recruits a large population of neurons and propagates like a wave over a wide region of the central nervous system. Here, we review spontaneous activity in the chick hindbrain by focusing on this large-scale synchronized activity. Asynchronous activity that is expressed earlier than the above mentioned synchronized activity and activity originating in midline serotonergic neurons are also briefly mentioned.

## Introduction

The developing nervous system generates spontaneous activity that is suggested to play a critical role in neural development (Moody and Bosma, 2005; Blankenship and Feller, 2010). Some of the earliest activity is expressed in the hindbrain and spinal cord. This spontaneous activity was originally considered to be a prototype of the rhythmic discharges in the adult central nervous system, such as respiratory and locomotor patterns. This classic view has been challenged by recent studies showing that the embryonic spontaneous activity has different characteristics of spatio-temporal patterns, origin, and pharmacological substrates from those of the adult central pattern generators (e.g., see the discussions of Chub and O’Donovan, 1998; Thoby-Brisson et al., 2005).

In past studies, spontaneous activity has often been analyzed using isolated hindbrains or spinal cords as these structures were considered to produce independent activities, which has caused some confusion. In fact, during early development activity propagates over a wide area of the central nervous system, maximally extending to the lumbosacral cord and forebrain, and thus most regions of the central nervous system are functionally correlated (Momose-Sato et al., 2001b, 2007, 2009, 2012a).

Here, we review spontaneous activity in the chick embryo by focusing on this large-scale synchronized activity. Much of what we learned about the synchronized activity has come from studies on the chick embryo because the embryo in an externally laid egg is readily available and amenable to surgical and pharmacological manipulation. Synchronized activity having characteristics similar to those described here has also been reported in the mammalian embryo, and we refer the reader to related reviews, as well as literatures focusing on the spinal cord (O’Donovan, 1999; Chatonnet et al., 2002; Marder and Rehm, 2005; Moody and Bosma, 2005; Greer et al., 2006; Hanson et al., 2008; O’Donovan et al., 2008; Bosma, 2010; Momose-Sato and Sato, 2013), which would be a great help for better understanding the present topic. In the last sections of this article, we also briefly mention asynchronous activity that is expressed earlier than the above mentioned synchronized activity, and activity originating in midline serotonergic neurons.

## Large-Scale Synchronized Activity

### Activity Pattern

The earliest studies of spontaneous activity in the chick embryo involve descriptions of embryonic motility observed *in ovo*. Preyer (1885) described this behavior more than a century ago, and it has been well characterized by Hamburger and Balaban (1963) (for reviews see Bekoff, 2001; Oppenheim and Lauder, 2001). Embryonic motility from stage 21 (E3.5, E: days of incubation in chicks) appears as periodically recurring sequences of slight flexions of the neck, with subsequently two or more S-waves extending from the head to the tail (Hamburger and Balaban, 1963). Direct evidence for the neurogenic basis of this behavior has been obtained from electrophysiological studies of spinal neuronal activity *in ovo*, which revealed a parallel between the electrical discharges and embryonic motility (Ripley and Provine, 1972; Provine, 1973).

**Figure 1:**
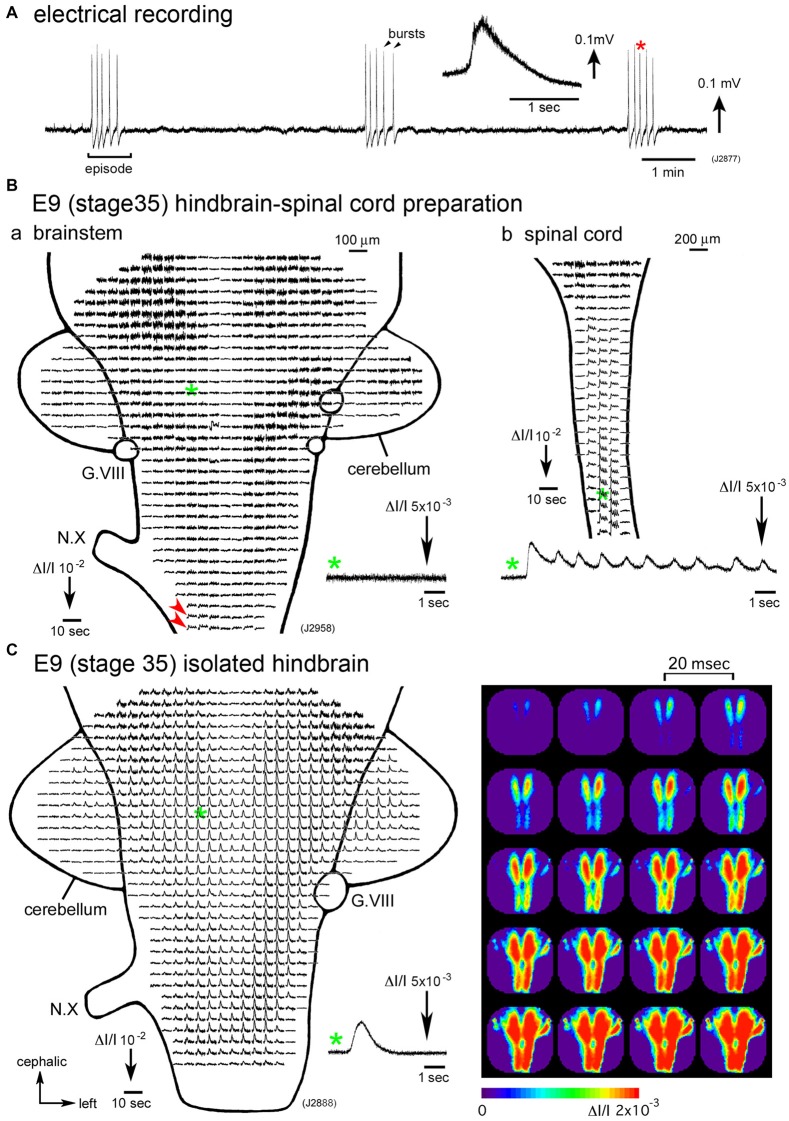
**(A)** Electrical recording of spontaneous activity in the chick embryo. The signal was recorded from a hindbrain-spinal cord preparation dissected from an E6 (stage 28) embryo with a glass micro-suction electrode applied to the root of the vagus nerve. In the inset, an enlarged trace of a single burst indicated with a red asterisk is presented. **(B)** Voltage-sensitive dye recording of the spontaneous activity in an E9 (stage 35) hindbrain-spinal cord preparation. Recordings in **(Ba,b)** were obtained from the hindbrain and cervical cord of the same preparation, respectively. Enlargements in optical signals indicated with green asterisks are presented in the lower side. Oscillatory activity was detected from the lower medulla (**Ba**, red arrowheads) and spinal cord **(Bb)**, but not in the pons (**Ba**, a green asterisk and inset), midbrain, or cerebellum. **(C)** Voltage-sensitive dye recording of the spontaneous activity that appeared in the isolated hindbrain at E9 (stage 35). A pseudo-color image on the right shows the propagation pattern of the spontaneous activity. The frame interval was 20 ms. Data shown in Figures 1B,C, 2 were obtained using a 1020ch optical recording system (Hirota et al., 1995; Momose-Sato et al., 2001a) with a voltage-sensitive dye, NK2761. The scale on the recordings and images indicates the fractional change in transmitted light intensity, ΔI/I. G.VIII, vestibulo-cochlear ganglion; N.X, vagus nerve (Reproduced from Mochida et al., 2009b; and Momose-Sato and Sato, 2014).

The cranial and spinal nerves show similar rhythmic bursting when the hindbrain and/or spinal cord is isolated *in vitro* (Landmesser and O’Donovan, 1984; Fortin et al., 1995; O’Donovan et al., 1998), indicating that motor outputs from these nerves produce embryonic motility. The activity is recorded as recurring episodes composed of several bursts, appearing at a low frequency with an inter-episode interval of a few minutes (Figure 1A). Similar spontaneous activity is detected in mouse and rat embryos (Nakayama et al., 1999; Abadie et al., 2000; Hanson and Landmesser, 2003; Ren and Greer, 2003; Momose-Sato et al., 2007, 2012a), indicating that this activity is globally generated across species.

Experiments manipulating the rhombomere (segmental structures of the early hindbrain) and rhombomere-specific genes have suggested that the expression of the burst is associated with the odd-numbered rhombomeres (r3 and r5) and their interaction with the adjacent even-numbered rhombomeres (Fortin et al., 1999), with some molecular cues expressed in rhombomere segments, such as *Krox20*, regulating the bursting pattern (Chatonnet et al., 2002; Borday et al., 2003; Coutinho et al., 2004). Although these experiments were performed in isolated hindbrains, in which the primary rhythm generator was deprived (see “Origin” Section), the results suggest that inter-segmental interactions are important for the generation of recurring bursting patterns.

### Synchronization

The spontaneous activity in the hindbrain and spinal cord is associated with the co-activation of different nerves/regions. Synchronization occurs between different rostrocaudal levels, between the left and right sides, and between the nerves innervating the flexor and extensor muscles (Provine, 1973; Fortin et al., 1994, 1995; Milner and Landmesser, 1999). Thus, the early spontaneous activity may be correlated with intersegmental and bilateral interactions between different neuronal subsets (Fortin et al., 1995). This has been visually demonstrated in optical studies using voltage-sensitive dyes, in which spontaneous as well as sensory-evoked waves spread over a wide region of the central nervous system, including the spinal cord, hindbrain, midbrain, cerebellum, and part of the forebrain (Momose-Sato et al., 2001b, 2007, 2009, 2012a). The propagation velocity of the wave was too slow to be attributed to axonal conduction along unmyelinated fibers at the corresponding stage (O’Donovan et al., 1994; Arai et al., 2007; Momose-Sato et al., 2007). The mechanisms underlying the spread of this activity may include the sequential synaptic activation of adjacent regions coupled by short-range synaptic connections (Fortin et al., 1995; O’Donovan et al., 2008), the non-synaptic release of transmitters and paracrine-like intercellular communication (Demarque et al., 2002; Scain et al., 2010), and the coordination of chemical transmitters with gap junctions, as well as electrical interactions between neighboring neurons (Hanson and Landmesser, 2003; Ren et al., 2006).

### Development

Correlated spontaneous discharges are recorded from stage 24 (E4) in the hindbrain (Fortin et al., 1994; Momose-Sato et al., 2009) and from stage 22.5–24 (E3.5–E4) in the spinal cord (Milner and Landmesser, 1999; Hanson and Landmesser, 2004). Marked changes occur in the activity patterns during development, such as an increase in the interval between episodes, the number of burst discharges within the episodes (a single burst to multiple bursts), and a decrease in the inter-burst interval within an episode (O’Donovan and Landmesser, 1987; Fortin et al., 1994; Milner and Landmesser, 1999).

Synchronization over the brain and spinal cord is observed during a particular period of development, E4–E8 (Momose-Sato et al., 2009; Momose-Sato and Sato, 2014). From E9 onward, spontaneous activity becomes segregated in the spinal cord, and the signal is very small or undetectable in the hindbrain (Momose-Sato and Sato, 2014; Figure 1B). The results indicate that the activity at E9 and later is no longer “large-scale”, but is specialized to the spinal network. Interestingly, spontaneous activity becomes detectable in the E9 hindbrain when the hindbrain is isolated, although no such activity is observed when the spinal cord is intact (Momose-Sato and Sato, 2014; Figure 1C). This seems due to a change in neural excitability in the hindbrain, which is caused by deprivation of the spinal rhythm generator (also see “Origin” Section).

In the mouse and rat embryos, it has been reported that large-scale synchronized activity is substituted by segregated activity in the caudal spinal cord and rostrolateral medulla, which seem to correspond to the locomotor and respiratory rhythm generator, respectively (Momose-Sato et al., 2012a). The underlying mechanism in the mouse involves the switching of γ-aminobutyric acid (GABA) acid responses from excitatory to inhibitory (Momose-Sato et al., 2012b). Nevertheless, this seems not to be the case in the chick embryo because the application of the GABA_A_ receptor antagonist bicuculline does not restore the activity in the E9 hindbrain (Momose-Sato and Sato, 2014) and even suppresses the activity in the spinal cord until at least E11 (Chub and O’Donovan, 1998). Possible mechanisms include the intrinsic excitability of neurons being decreased with hyperpolarization of the resting membrane potential or other changes in electrical properties, and also weakened functional connections in neurons under the level required to mediate the conduction of the activity. It is also possible that some inhibitory signals are derived from ascending fibers or targets innervated by descending fibers, or the effects of neuromodulators (Whelan, 2003; Marder and Rehm, 2005).

### Origin

**Figure 2:**
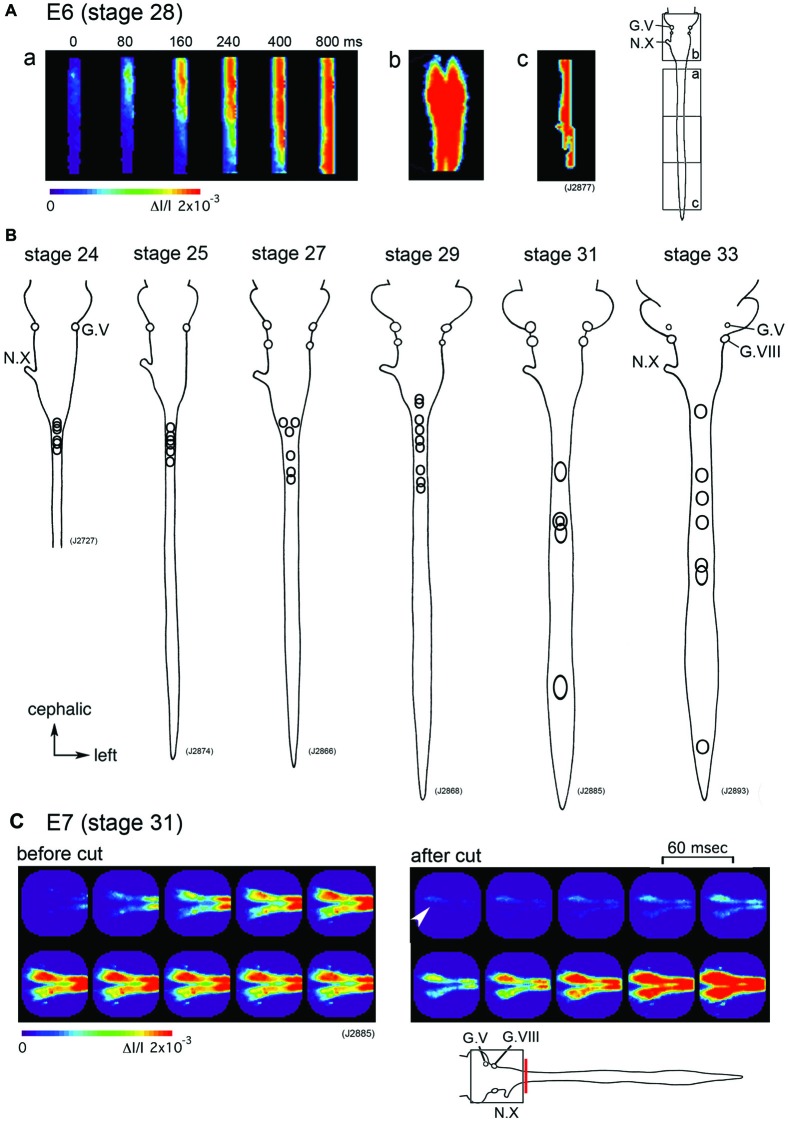
**(A)** The propagation pattern of the spontaneous activity in an E6 (stage 28) hindbrain-spinal cord preparation. The activity initiated in the upper cervical cord **(a)**. Images **(Ab,c)** present maximum responses in the hindbrain and caudal cord, respectively. Images were obtained from the hindbrain and spinal cord indicated with squares in the right inset. **(B)** The origins of the spontaneous activity are indicated with circles for the most typical preparation at stage 24–33. In each preparation, one circle corresponds to one spontaneous wave, and variations in circle locations show variations in the origin of the activity. **(C)** Pseudo-color images of the spontaneous activity in an E7 (stage 31) hindbrain-spinal cord preparation before (left images) and after (right images) the obex was cut. Images were obtained from the hindbrain region indicated with a square in the lower inset. The red vertical line shows the location where the cut was made. An arrowhead in the right image indicates the origin of the activity. The frame interval was 60 ms. G.V, trigeminal ganglion; G.VIII, vestibulo-cochlear ganglion; N.X, vagus nerve (Reproduced from Momose-Sato et al., 2009; and Momose-Sato and Sato, 2014).

Which part of the central nervous system functions as a generator of the large-scale synchronized activity? An optical imaging study using hindbrain-whole spinal cord preparations have shown that the origin of the spontaneous wave is in the upper cervical cord/lower medulla at stage 24–29 (E4–E6) with some variations between the activities (Figures 2A,B). As development proceeds to stage 30–34 (E7–E8), the region responsible for generating the wave shifts caudally, and the activity is initiated in any part of the spinal cord (Momose-Sato et al., 2009; Figure 2B).

Although spontaneous activity is typically initiated in the spinal cord in intact preparations, activity can be detected in the isolated hindbrain, in which the primary rhythm generator is deprived (Fortin et al., 1995, 1999; Momose-Sato and Sato, 2014). Furthermore, this rhythmic pattern is preserved in transverse sections of the hindbrain (Fortin et al., 1995). Moreover, the spontaneous activity is generated by single rhombomeres when isolated at E2 (stage 10–11; Fortin et al., 1999; Borday et al., 2003). Thus, neurons and/or neuronal networks producing spontaneous activity are widely distributed in the hindbrain and spinal cord, with a specific excitable region probably pacing the activity in intact preparations. When the primary pacing area is removed, as is the case for the isolated hindbrain, other potential generators become initiators of spontaneous activity, and produce activity with a similar pattern to the previous one (Momose-Sato et al., 2007; Momose-Sato and Sato, 2014; Figure 2C). This homeostatic compensation is an active process, which is associated with an increase in excitability and/or the number of neurons recruited to the activity (Momose-Sato and Sato, 2014).

According to the model proposed in the chick spinal cord, the generation of spontaneous activity is dependent on the degree of neuronal connectivity and the number of units recruited to the wave. The activity is not generated in a particular class of cells, but is initiated where the connection is sufficient enough to sustain the propagation of activity throughout the whole network (Chub and O’Donovan, 1998; O’Donovan et al., 1998). A similar model might be applicable to the neural network in the hindbrain.

### Pharmacology

Nicotinic acetylcholine receptors, specifically those without the α7-subunit, mediate spontaneous synchronized activity at early developmental stages (stage 25–28: E4.5–E6), and glutamate receptors at later stages (stage 33~: E8~; Chub and O’Donovan, 1998; Milner and Landmesser, 1999; Hanson and Landmesser, 2004; Mochida et al., 2009b). GABA and glycine also act as excitatory mediators, most likely because the Cl^−^ reversal potential (E_Cl_) is more positive than the resting potential, which is caused by the high intracellular Cl^−^ concentration (Chub and O’Donovan, 2001).

In mouse and rat embryos, it has been reported that spontaneous activity undergoes two types of developmental changes in pharmacological substrates. One is a switching of the dominant contributor from nicotinic acetylcholine receptors to glutamate receptors, and the other is the change in GABA/glycinergic responses from depolarizing/excitatory to hyperpolarizing/inhibitory (Nakayama et al., 1999; Ren and Greer, 2003; Myers et al., 2005; Ladle et al., 2007; Momose-Sato et al., 2012b). In the chick embryo, the dominant neuronal response to GABA and glycine is depolarizing/excitatory at least until E8 in the hindbrain (Momose-Sato et al., 1998) and E10–E11 in the spinal cord (Chub and O’Donovan, 1998; Gonzalez-Islas and Wenner, 2006). On the other hand, some neurons in the hindbrain reticular formation receive hyperpolarizing inputs from GABAergic neurons, which seem to regulate the high-frequency bursts of the spontaneous activity (Fortin et al., 1999).

In addition to chemical synaptic antagonists, synchronized activity is inhibited by putative gap junction blockers such as octanol, carbenoxolone, and 18ß-glycyrrhetinic acid (Milner and Landmesser, 1999; Mochida et al., 2009b). Although interpretation of the results gained using these blockers is not forthcoming because of the non-specific effects of the drugs on cell membrane conductance, a recent study in the mouse spinal cord demonstrated that 18ß-glycyrrhetinic acid and meclofenamic acid exhibited specific effects on gap junctions (Czarnecki et al., 2014), suggesting that the correlated activity is mediated by the coordination of chemical neurotransmitter systems and gap junctional communication.

## Asynchronous Activity Expressed Earlier than the Synchronized Activity

In addition to the synchronized activity discussed above, asynchronous excitation has been detected in the chick hindbrain using Ca^2+^-imaging (Mochida et al., 2009a). In this study, both retrogradely labeled reticulospinal and vestibuloocular neurons exhibited asynchronous transients earlier than the emergence of synchronized activity in each population (stage 25 in the reticulospinal neurons and stage 26 in the vestibuloocular neurons). The asynchronous and synchronous activities were considered to be independent phenomena produced by different mechanisms since: (1) the asynchronous activity was not inhibited by tetrodotoxin, which blocks the synchronous activity; and (2) there is no temporal or developmental relationship between the two activities (Mochida et al., 2009a). Thus, in the early stages of development, the activity of individual neurons is independent, but later becomes synchronous. A similar developmental sequence of spontaneous activity occurs in the mouse hindbrain (Abadie et al., 2000; Gust et al., 2003) and other brain regions (for a review see Allene and Cossart, 2010).

## Midline Spontaneous Activity

Hughes et al. (2009) reported spontaneous activity that arises in the midregion of the hindbrain and travels in both the rostral and caudal directions along the midline. This activity resembled the midline spontaneous activity reported in the mouse embryo, which is produced by serotonergic neurons (for a review see Bosma, 2010). In the chick, spontaneous waves that originate in the spinal cord usually precede the midline activity (Hughes et al., 2009), suggesting that it occurs secondarily following the synchronized activity.

## Future Perspectives

Recent advances in electrophysiology, molecular biology, and optical imaging have shed much light on our understanding of the widely-propagating synchronized activity in the hindbrain and spinal. Despite much knowledge on the global features of this activity, many unanswered questions remain. Perhaps the most important issue is the functional significance of the activity. Primordial activity in the spinal cord has been suggested to play a significant role in developmental processes, including axon pathfinding (Hanson and Landmesser, 2004; Hanson et al., 2008) and establishment of locomotor function (Myers et al., 2005). On the other hand, investigations in the hindbrain are less advanced. Spontaneous synchronized activity is observed in several systems of the developing brain, and it is suggested to play an instructive role in the synaptic network formation (Zhang and Poo, 2001; Kirkby et al., 2013; Andreae and Burrone, 2014). Synchronized activity in the chick hindbrain is only expressed within a restricted period, E4–E8, during which functional synaptic connections are established in the brainstem nuclei (Momose-Sato et al., 2001a; Glover et al., 2008; Momose-Sato and Sato, 2011). One challenge for the future would be to ascertain whether this process is under the control of spontaneous activity in the hindbrain.

## Author Contributions

YM-S designed the research. YM-S and KS performed experiments, analyzed data, and prepared the manuscript.

## Conflict of Interest Statement

The authors declare that the research was conducted in the absence of any commercial or financial relationships that could be construed as a potential conflict of interest.

## Abbreviations

E: embryonic day, which shows days of incubation in chicks
GABA: γ-aminobutyric acid.
